# A case series of delayed primary closure of abdominal laparotomy incisions after perforated diverticulitis

**DOI:** 10.1093/jscr/rjaf825

**Published:** 2025-10-17

**Authors:** Autumn D Pak, Isabel M Kiko, Elisabeth A Loomis, Katie Francis

**Affiliations:** Department of General Surgery/Trauma, Akron City Hospital, Akron City Hospital Summa Health, 55 Arch Street, Suite 2F, Akron, OH 44304, United States; Northeast Ohio Medical University, Northeast Ohio Medical University, 4209 St. Rt. 44, PO Box 95, Rootstown, Ohio 44272, United States; Department of General Surgery/Trauma, Akron City Hospital, Akron City Hospital Summa Health, 55 Arch Street, Suite 2F, Akron, OH 44304, United States; Northeast Ohio Medical University, Northeast Ohio Medical University, 4209 St. Rt. 44, PO Box 95, Rootstown, Ohio 44272, United States; Department of General Surgery/Trauma, Akron City Hospital, Akron City Hospital Summa Health, 55 Arch Street, Suite 2F, Akron, OH 44304, United States; Northeast Ohio Medical University, Northeast Ohio Medical University, 4209 St. Rt. 44, PO Box 95, Rootstown, Ohio 44272, United States; Department of General Surgery/Trauma, Akron City Hospital, Akron City Hospital Summa Health, 55 Arch Street, Suite 2F, Akron, OH 44304, United States; Northeast Ohio Medical University, Northeast Ohio Medical University, 4209 St. Rt. 44, PO Box 95, Rootstown, Ohio 44272, United States

**Keywords:** delayed primary closure, temporary abdominal closure, surgical site infections

## Abstract

The management of contaminated abdominal incisions after surgery for perforated hollow viscus organs remains controversial. Limited studies have shown some benefits to delayed primary closure over primary closure, specifically related to a decrease in surgical site infections (SSI) and decreased length of stay; however, there is little research looking at its effect on decreasing outpatient wound care needs*.* The three patients successfully underwent delayed primary closure of their midline incisions after exploratory laparotomy for perforated diverticulitis. None required additional wound care in the form of home health, wound care clinic visits, or nursing facility placement after discharge. All three patients did not develop any SSIs postoperatively. This case series demonstrated that delayed primary closure with the aid of negative pressure wound therapy is an effective way to minimize outpatient wound care needs and costs and is effective at managing contaminated abdominal incisions.

## Introduction

The management of contaminated abdominal incisions after surgery for perforated hollow viscus organs remains controversial. Patients have a high rate of developing surgical site infections (SSIs) after primary closure; however, wounds that heal by secondary intention carry their own morbidity with prolonged healing times requiring long-term and costly wound care. Delayed primary closure (DPC), historically, has been used as a compromise between these two options [1–6].

Small and mostly retrospective studies [2–5] have shown some benefits to DPC over primary closure, specifically related to a decrease in SSIs and decreased length of stay (LOS); however, there is little research looking at its effect on decreasing outpatient wound care needs. We present three different cases of perforated diverticulitis with contaminated laparotomy incisions that underwent DPC with successful results.

## Methods and results

### Patient A

**Figure 1:**
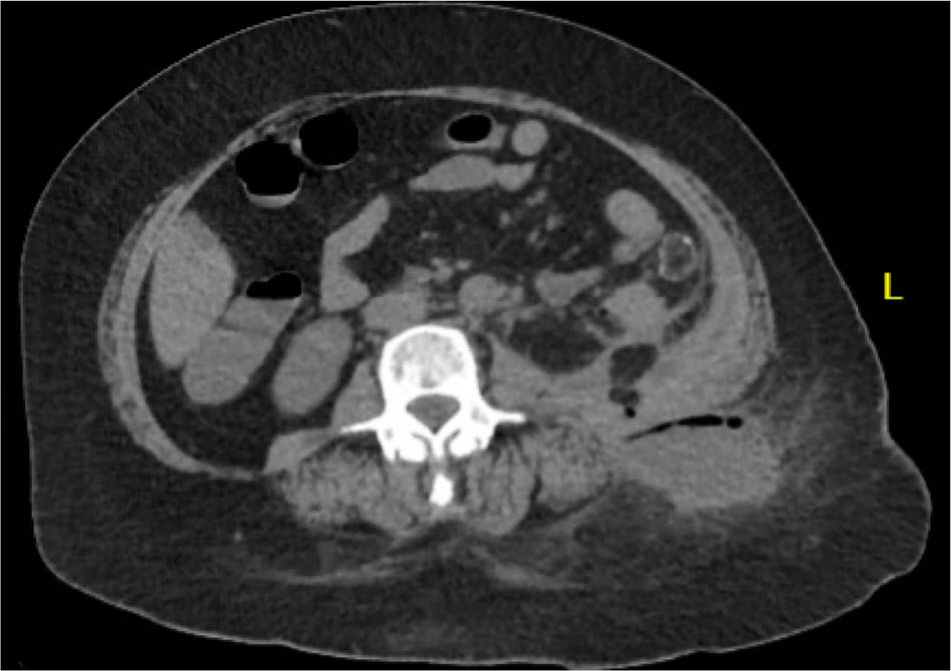
Patient A. Initial CT abdomen and pelvis showing left subcutaneous fluid collection extending intra-abdominally.

**Figure 2:**
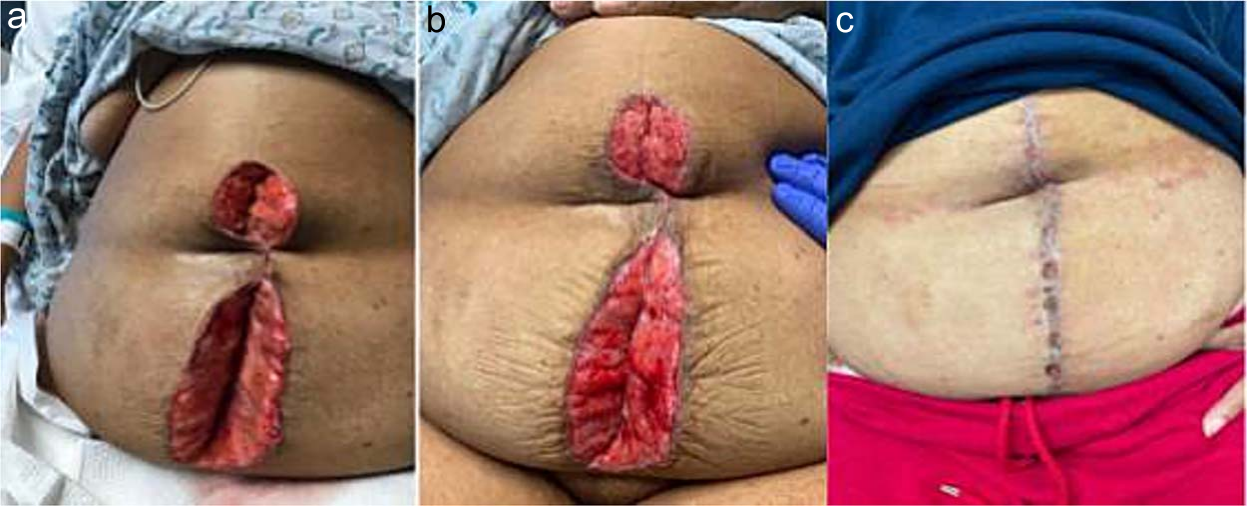
Patient A. a. Incision at first wound vac change after initial operation (POD 3). b. Incision prior to DPC (POD 13). c. Incision at first post op visit (15 days after DPC).

A 64 year old female with a history of recent diverticulitis treated with outpatient antibiotics and a fall two months prior to her left flank presented to the hospital with persistent left flank and abdominal pain. Initial workup showed a left flank fluid collection that connected to the left psoas muscle and retroperitoneum with associated descending/sigmoid diverticulosis (Fig. 1). The patient was admitted, started on empiric IV antibiotics, and an IR-guided drain was placed into the subcutaneous abscess. A repeat computed tomography (CT) scan done with rectal contrast showed a colocutaneous fistula to the descending/sigmoid colon in the area of the previous abscess. Therefore the patient was taken to the operating room on hospital day two. Intra-operatively, the patient was found to have purulent peritonitis, left colon diverticulitis, a colocutaneous fistula, and an associated pericolic abscess. The patient underwent extensive lysis of adhesions, take down of fistulous tract, and left colectomy with primary anastomosis. The fascia was closed with #1 non looped PDS and interrupted figure of eight stitches. The umbilicus was reapproximated with sutures and a negative pressure subcutaneous wound vac was placed to the midline laparotomy incision and kept on continuous suction at -125mmhg. The first wound vac change occurred on postoperative day (POD) 3 (Fig. 2a) and then the wound vac was changed every other day in the hospital until a layer of granulation tissue was formed along the entire incision. On POD 13, DPC was performed by reapproximating the skin edges with staples (Fig. 2b). The patient’s hospital course was complicated by a prolonged postoperative ileus requiring supplemental total parenteral nutrition (TPN). The patient was discharged to home on POD 13 tolerating a diet and with one week of oral antibiotics. The patient was then seen in the office 15 days after discharge and DPC on POD 28. The midline incision was closed, healing well, and was without any signs of SSI (Fig. 2c).

### Patient B

**Figure 3:**
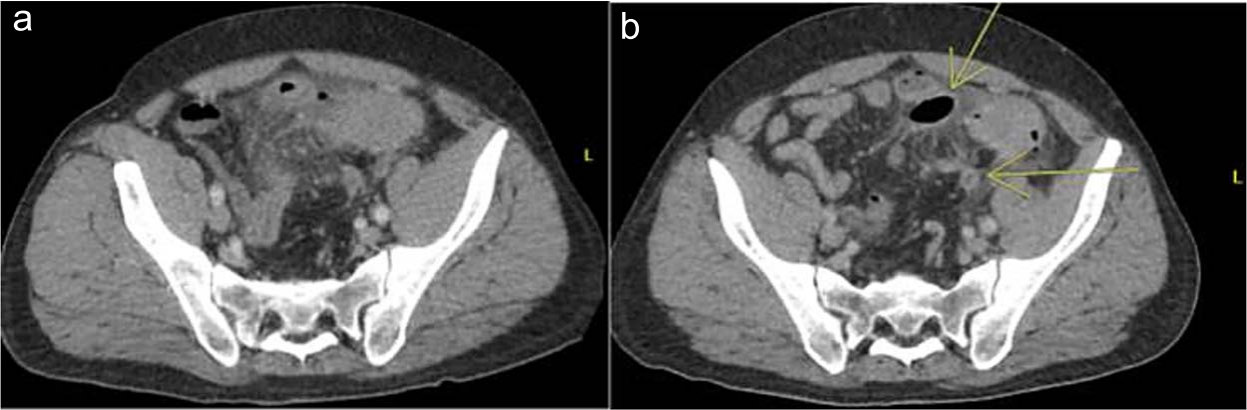
Patient B. a. Initial CT abdomen and pelvis showing acute diverticulitis with small adjacent abscess. b. Repeat CT abdomen and pelvis with worsening complicated diverticulitis.

**Figure 4:**
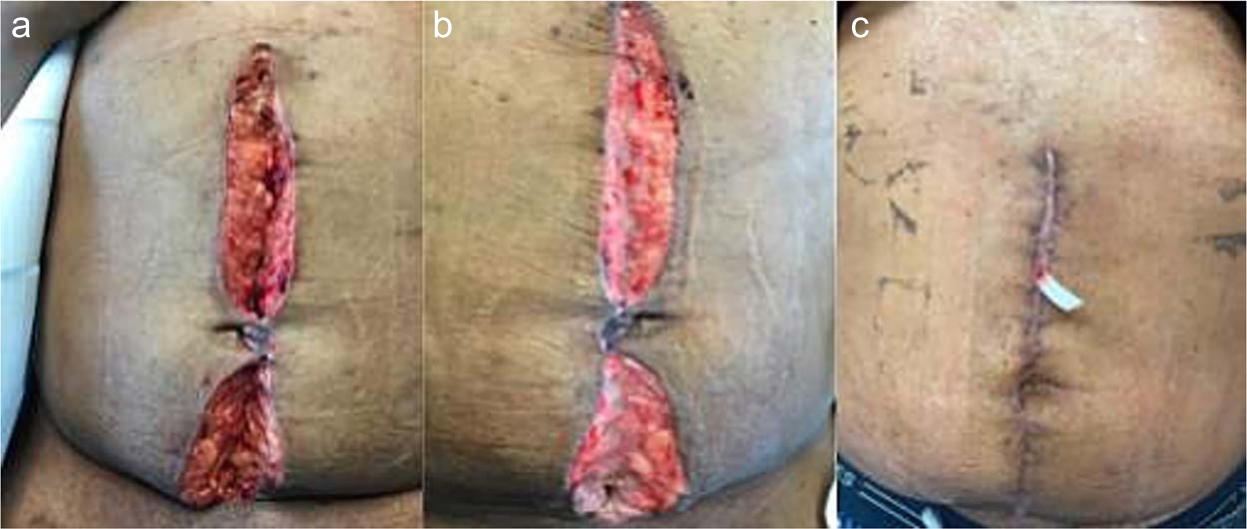
Patient B. a. Incision at first wound vac change after initial operation (POD 2). b. Incision prior to DPC (POD 8). c. Incision at first post op visit (27 days after DPC).

A 44 year old male presented to the hospital with left lower quadrant abdominal pain and diarrhea. Initial workup showed sigmoid diverticulitis with a small pericolic abscess (<3 cm) (Fig. 3a). The patient was admitted to the hospital and treated with bowel rest and IV antibiotics. Unfortunately, the patient failed nonoperative management and repeat CT imaging showed increased extraluminal air (Fig. 3b). He was taken to the operating room on hospital Day 8. Intra-operatively, the patient was found to have purulent peritonitis, left colon diverticulitis, and associated pericolic abscess. He underwent a left colectomy with primary anastomosis. Fluid cultures were obtained. The fascia was closed with #1 non looped PDS and interrupted figure of eight stitches. The umbilicus was approximated with sutures, and a negative pressure subcutaneous wound vac was placed to the midline laparotomy incision and kept on continuous suction at -125 mm Hg. The first wound vac change occurred on POD 2 (Fig. 4a) and then the wound was changed every other day similar to Patient A. DPC was performed on POD 8 (Fig. 4b). The patient’s hospital course was complicated by a prolonged postoperative ileus requiring TPN and an upper GI bleed that was treated medically with PPI therapy. The patient did also have a postoperative abscess that was treated with an IR-guided drain and prolonged antibiotic therapy until POD 20. The patient’s stay was further prolonged due to his initial need for a nursing facility at discharge- he was from out of state and his insurance was not accepted at any facilities. He was ultimately discharged to home on POD 20. The patient was then seen in the office 15 days after discharge and 27 days after DPC on POD 35. The midline incision was healing well and was without any signs of SSI (Fig. 4c).

### Patient C

**Figure 5:**
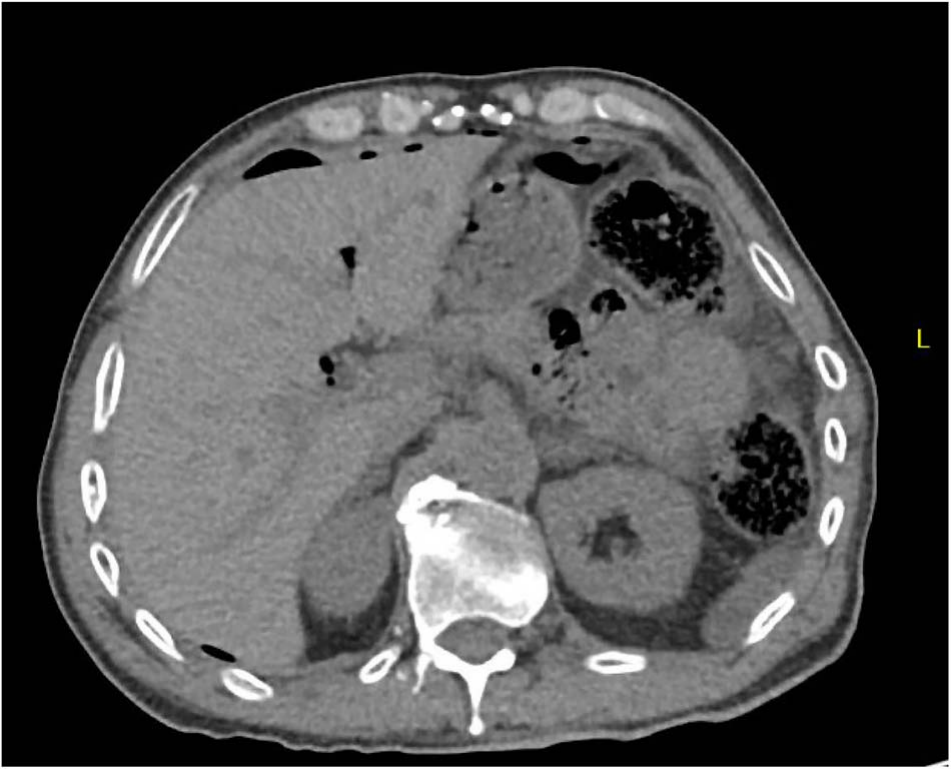
Patient C. Initial CT abdomen and pelvis showing free air concerning for perforated hollow viscus.

**Figure 6:**
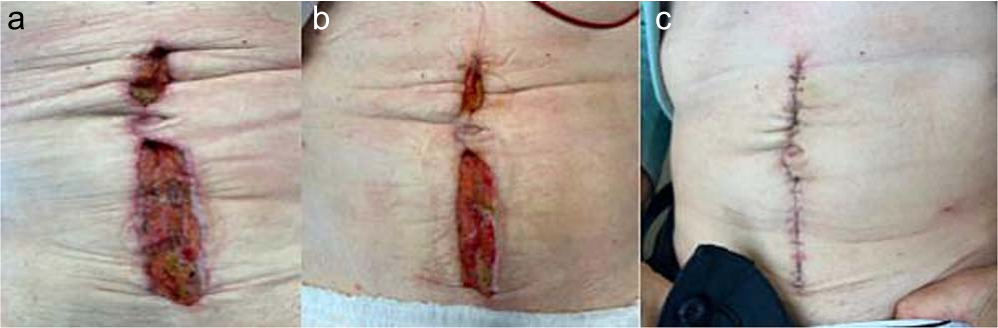
Patient C. a. Incision at first wound vac change after initial operation (POD 4). b. Incision prior to DPC (POD 11). c. Incision at first post op visit (10 days after DPC).

A 71 year old male presented to the hospital with abdominal pain and free air on initial CT scan (Fig. 5). Of note, the patient had a recent right total hip replacement 2 days prior by the orthopedic surgery team. The patient was taken emergently to the operating room and was found to have perforated sigmoid diverticulitis with feculent peritonitis. He underwent a left colectomy with primary anastomosis. The fascia was closed with #1 non looped PDS and interrupted figure of eight stitches. The umbilicus was reapproximated with sutures, and a negative pressure subcutaneous wound vac (black foam) was placed to the midline laparotomy incision and kept on continuous suction at -125mm Hg. The first wound vac change occurred on POD 4 (Fig. 6a) and then the wound was changed every other day similar to the above patients. DPC was performed on POD 11 (Fig. 6b). The patient’s intraoperative cultures grew *E. coli* and pseudomonas and he completed a 10 day course of antibiotics. The patient was discharged to acute rehab on POD 11. The patient was then seen in the office 10 days after discharge and DPC on POD 21. The midline incision was healing well and was without any signs of SSI (Fig. 6c).

## Discussion

In this case series, we demonstrate three patients with successful delayed primary closure of their laparotomy incisions without complications such as SSIs or prolonged LOS due to wound complications. Additionally, the use of DPC allowed for decreased wound care needs as an outpatient and was beneficial specifically to the two patients that did not have medical insurance to cover outpatient wound care costs. Our cases showed that delayed primary closure can be an effective method, both clinically and financially, to manage contaminated abdominal incisions in the setting of perforated diverticulitis. However, this was a limited case series and additional investigation should be undertaken to further explore the clinical and statistical significance that DPC has on management of infected abdominal incisions.
